# Study of psychosocial factors affecting premature ejaculation from the perspective of personality traits: a large sample cross-sectional study from Anhui, China

**DOI:** 10.1093/sexmed/qfaf094

**Published:** 2025-11-15

**Authors:** Jingjing Gao, Rui Gao, Kun Wang, Guosheng Xu, Zifang Zhang, Xiaodong Bai, Jishuang Liu, Kai Shi, Jun Mao, Yao Zhang, Xiansheng Zhang

**Affiliations:** Department of Urology and Andrology, The First Affiliated Hospital of Anhui Medical University, Hefei, 230000, China; Department of Urology and Andrology, The First Affiliated Hospital of Anhui Medical University, Hefei, 230000, China; Department of Urology and Andrology, The First Affiliated Hospital of Anhui Medical University, Hefei, 230000, China; Department of Urology and Andrology, The Hefei No. 1 People's Hospital, Hefei, 230000, China; Department of Urology and Andrology, The First Affiliated Hospital of Anhui Medical University, Hefei, 230000, China; Department of Urology and Andrology, The Luan No. 4 People's Hospital, Luan, 237000, China; Department of Urology and Andrology, The Anqing Municipal Hospital, Anqing, 246000, China; Department of Urology and Andrology, The Affiliated Suzhou Hospital of Anhui Medical University, Suzhou, 234000, China; Department of Urology and Andrology, The Affiliated Yijishan Hospital of Wannan Medical College, Wuhu, 240000, China; Department of Urology and Andrology, The First Affiliated Hospital of Anhui Medical University, Hefei, 230000, China; Department of Urology and Andrology, The First Affiliated Hospital of Anhui Medical University, Hefei, 230000, China

**Keywords:** premature ejaculation, personality traits, psychosocial factors, Myers-Briggs Type Indicator

## Abstract

**Background:**

Personality traits are the intrinsic factors of mental health, and may play a role in the pathogenesis of premature ejaculation (PE), but few studies have evaluated the association between personality traits and the 4 types of PE.

**Aim:**

We aim to investigate the personality traits associated with PE in 4 types of PE and their associations with PE.

**Methods:**

Between February 2024 and February 2025, we conducted a survey in Anhui, China, and enrolled 1708 males. Personality traits were independently assessed by the Myers-Briggs Type Indicator (MBTI). The index of PE was used to evaluate ejaculation control, sexual life satisfaction, and distress caused by PE.

**Outcomes:**

Energy-Introversion, Perceiving-Sensing, Judging-Feeling, and Orientation-Perception may influence men’s sexual activity, and the Energy, Perceiving, and Orientation have different effects on the 4 types of PE.

**Results:**

The ISFP personality type is most common in those with PE. Among the 4 subtypes of PE, the Introversion-Intuition-Feeling-Perceiving (INFP) personality type is most common in those with Lifelong PE (LPE), the Extraversion-Sensing-Feeling-Judging (ESFJ) personality type is most common in those with Acquired PE (APE), the Introversion-Sensing-Feeling-Perceiving (ISFP) personality type is most common in those with Variable PE (VPE), and the Extraversion-Sensing-Feeling-Perceiving (ESFP) personality type is most common in those with Subjective PE (SPE).

**Clinical Implications:**

Personality trait factors may influence the pathogenesis of PE, and the use of the MBTI personality assessment provides a new perspective on how personality traits play a role in the onset of PE.

**Strengths & Limitations:**

It is necessary to conduct larger-scale, more rigorous community-based studies to further elucidate the role of personality traits in the development of PE.

**Conclusion:**

Men with traits of Introversion, Sensing, Feeling, and Perception tend to have high sensitivity, introspection, and a deep pursuit of idealized relationships.

## Introduction

Premature ejaculation (PE) is a common male sexual dysfunction and is also recognized as a physical and psychological disease in men. Most studies have confirmed that the onset of PE is closely related to mental and psychological factors.^1^ According to the definition of PE endorsed by the Italian Society of Andrology and Sexual Medicine (SIAMS), the diagnosis is “male sexual dysfunction characterized by ejaculation that always or nearly always occurs prior to or within approximately 1 min of vaginal penetration from the first sexual experience (lifelong premature ejaculation), or a clinically significant reduction in latency time, often to approximately 3 min or less (acquired premature ejaculation); the inability to delay ejaculation on all or nearly all vaginal penetrations; and negative personal consequences, such as distress, bother, frustration, and/or the avoidance of sexual intimacy.”^2^^,^^3^ For example, a negative mental state may lead to the onset of PE, and the sexual disharmony caused by PE can further exacerbate negative emotions.^4^

Premature ejaculation is caused by the combined effects of multiple factors. It may be associated with various conditions such as hyperthyroidism, chronic pelvic pain syndrome, varicocele of the spermatic cord, and impotence.^5^ Furthermore, the SIAMS has noted that behavior and attitudes may affect the ability to control ejaculation, and that standardized psychological measurement tools can be used to assess the factors related to PE.^3^ Personality traits, as intrinsic factors of mental health, may play a role in the onset of PE. Research on the relationship between personality traits and the onset of PE will have significant clinical and social implications for further understanding how mental health factors influence the pathogenesis of PE.

Personality traits, as internal factors influencing an individual’s mental and psychological activities, typically refer to a relatively stable emotional feedback that is exhibited in some aspects such as emotions, cognition, and behavior. These traits are closely related to an individual’s mental health status.^6^ For example, men with lower emotional stability are more likely to experience anxiety and depression when facing difficulties, whereas extroverted men generally exhibit greater psychological resilience and social adaptability. Therefore, this personality may enable men to have better self-regulation abilities and a more positive sexual attitude, leading to greater sexual satisfaction.

The Myers-Briggs Type Indicator (MBTI) is a personality assessment tool based on the psychological type theory of the Swiss psychologist Carl Jung that has been widely applied in areas such as career planning, team building, and personal growth.^7^ This assessment tool primarily reflects individual personality preferences through a series of self-reported questions. It has undergone extensive validity and reliability studies since its inception, and is generally considered to have good validity and reliability. Myers-Briggs Type Indicator divides personality into 4 dimensions, and each dimension is further divided into 2 opposing inner traits: Extraversion (E) and Introversion (I), Sensing (S) and Intuition (N), Thinking (T) and Feeling (F), as well as Judging (J) and Perceiving (P). The interplay and combination of these inner traits help researchers better understand an individual’s behavioral patterns, communication styles, and decision-making methods, which are crucial for the precise analysis and positioning of personality. Notably, the repeatability of MBTI is relatively poor. However, our main focus is on the correlation between personality traits and PE. The MBTI has been widely used in personality trait surveys and thus has a certain degree of reliability.

At present, this is the first study to investigate personality traits and PE onset on the basis of the MBTI assessment tool, which will provide a new perspective for further revealing the mechanism of personality traits in PE onset.

## Materials and methods

**Study population**

This study is a large-scale, multicenter cross-sectional sampling study conducted in Anhui, China. The study period spans from February 2024 to February 2025. The study population primarily included men with self-reported PE and healthy controls. The study participants in the PE group mainly consisted of male patients who visited urology clinics due to self-reported PE, whereas those in the healthy control group primarily included men who underwent health checks and were not diagnosed with PE.

To ensure the accuracy of the research and meet its objectives, the study samples must satisfy the following conditions: (1) be at least 18 years old and have a stable sexual life history with the opposite sex for over 6 months; (2) possess basic language skills and be able to complete the assessment questionnaire according to the instructions provided; (3) have normal erectile function; (4) have some questions in the assessment questionnaire about evaluating mental and psychological activities to ensure the accuracy of the responses, and the study population must not have suffered from severe mental diseases (such as schizophrenia, mood disorders, etc.); and (5) currently not taking any medications that may affect ejaculation function (such as serotonin reuptake inhibitors).

**Research process**

All participants provided written informed consent prior to their participation, ensuring that they were fully aware of the study’s purpose and procedures. The content of this study was reviewed and approved by the Ethics Committee of the hospital (No. 20190015). The research process is mainly divided into 3 main stages: design, implementation, and summary. During the design phase, since the study type was a multicenter sampling survey located at the hospital, the selection of cities was based on the geographical, population, and economic distribution of Anhui, China. Five cities (Huaibei, Wuhu, Anqing, Luan, and Hefei) were chosen to represent the northern, southern, eastern, western, and central regions of Anhui. Moreover, before the survey questionnaire entered the implementation phase, preliminary research with a small sample size (*N* = 30) was conducted to refine and revise the relevant questions and answers, ensuring the accuracy and smoothness of the research.

The implementation of the study is mainly divided into the following 4 stages, each specifically implemented by male physicians who have undergone unified research training: (1) the first step involves collecting basic information about the study population, primarily including age, education level, occupation, and place of residence; (2) the second step involves gathering information on the sexual history, medical history, and medication use of the study population; (3) the third step involves assessing the PE condition of patients, which includes the diagnosis and classification of PE, IELT, and the Chinese version of the IPE questionnaire; and (4) the fourth stage involves evaluating the personality traits of the study population via the MBTI personality questionnaire.

The diagnosis of PE subtypes is primarily based on the definitions of 4 PE subtypes provided by ISSM, which are classified as lifelong PE, acquired PE, variable PE, and subjective PE (for specific definitions of PE, see Figure SS1).^2^ However, it is necessary to emphasize that there is another situation where ED and PE can coexist in the same patient, namely, loss of control over both erection and ejaculation (less control of erection and ejaculation, LCEE).^8^ However, there are currently few studies related to LCEE, and since this is an exploratory study, our main focus remains on investigating the correlation between personality traits and PE by starting from the 4 main types of PE as defined in the ICD-11. We must emphasize that the classification of “four types of PE” is a viewpoint-based classification system, and this classification framework is currently being revised by academic organizations such as the ISSM.^9^ The IPE questionnaire is an assessment tool used to quantify the subjective symptoms and impact of PE, covering 3 symptom dimensions: ejaculation control ability, sexual satisfaction, and psychological distress.^10^ The answers to specific questions range from “never” to “always,” with a total score ranging from 0 to 50. A lower total score indicates more severe symptoms of PE. The MBTI personality questionnaire is a widely used tool in psychology research and clinical practice for assessing personality traits, helping us gain a more comprehensive understanding of individual behavior patterns, thought processes, and emotional responses.^11^ In this study, based on the basis of the assessment attributes of the MBTI personality questionnaire, we summarized the characteristics of each dimension’s inherent traits: (1) Extraversion and Introversion: Extraverted individuals tend to draw energy from the external world, enjoying social interactions. Introverted individuals, on the other hand, draw energy from their inner world, preferring solitude or deep conversations with a few people; (2) Sensing and Intuition: Sensing individuals focus on reality and concrete information, relying on the 5 senses to perceive the world. Intuitive individuals pay more attention to future possibilities, abstract concepts, and underlying meanings; (3) Thinking and Feeling: Thinking individuals prioritize logic and objective analysis when making decisions. Emotional types place greater emphasis on interpersonal relationships and emotional factors; (4) Judging and Perceiving types: Judging types prefer a planned and organized lifestyle, tending to make quick decisions. Perceiving types are more flexible and open, enjoying keeping options open. The internal consistency values of the Chinese versions of the IPE and MBTI assessment questionnaires in this study were 0.78 and 0.82, respectively.

### Statistical analysis

The organization and analysis of the research data were conducted using the SPSS 20.0 software. The descriptive statistics for the quantitative data are presented primarily as the means ± standard deviation, whereas the qualitative data are mainly as numbers (percentages). When the self-reported PE group data were compared with the control group data, the intergroup comparisons of the quantitative data were mainly conducted via independent samples *t*-tests, and the intergroup comparisons of the qualitative data were mainly conducted via chi-square tests. When 4 subtypes of PE were compared, intergroup comparisons of quantitative data were mainly conducted via ANOVA, and intergroup comparisons of qualitative data were mainly conducted via chi-square tests. To analyze the correlation between the MBTI score and the IELTS score and IPE, a partial correlation analysis model was used after adjusting for age effects. In this study, when the statistical indicator *P* < .05, the differences between the data were considered statistically significant. Moreover, we performed False Discovery Rate (FDR) correction for all the samples.

## Results

### Demographic information of males with and without PE complaints

A total of 1708 males completed the entire study, and the completion rate was approximately 71% (1708/2398). Among those who did not complete the study, 194 people lost contact, 78 people quit midway, 303 people completed incomplete questionnaires, and 115 people had other reasons.

A total of 669 (39.17%,669/1708) men reported PE and 1009 (59.07%,1009/1708) men did not report PE. The average age of the self-reported PE group was 40.72 ± 13.36 years, whereas that of the non-PE group was 33.37 ± 15.75 years.

Among the PE subtypes, LPE accounted for 19.28% (129/669), APE accounted 40.66% (272/669), VPE accounted 17.79 (119/669), and SPE accounted 22.27% (149/669). According to the age distribution, the average age of the VPE group was 32.29 ± 11.02 years, whereas that of the APE group was 47.75 ± 15.33 years. Other demographic characteristics of the study population are detailed in Table 1.

**Table 1:** Demographic information of males with and without PE complaints.

Demographic information	With PE complaints(N = 669)	Without PE complaints(N = 1009)	*P* ^1^	LPE (N = 129)	APE (N = 272)	VPE (N = 119)	SPE (N = 149)	*P* ^2^
**Age, years**	40.72 ± 13.36	33.37 ± 15.75	*<0.001*	35.82 ± 12.87	47.75 ± 15.33	32.29 ± 11.02	38.84 ± 10.42	*<0.001*
**BMI score, kg/m^2^**	24.38 ± 4.35	22.56 ± 3.85	*<0.001*	23.35 ± 4.02	25.82 ± 3.91	23.47 ± 4.15	23.37 ± 3.82	*<0.001*
**Smoking, n (%)**	402 (60.09%)	471 (46.68%)	*<0.001*	77 (59.69%)	197 (72.43%)	63 (52.94%)	65 (43.62%)	*<0.001*
**Exercise, n (%)**	258 (38.57%)	437 (43.31%)	*0.01*	50 (38.76%)	78 (28.68%)	56 (47.06%)	74 (49.66%)	*<0.001*
**Educational status, n (%)**			*0.44*					*0.41*
*High school or less*	212 (31.69%)	302 (29.93%)		45 (34.88%)	78 (28.68%)	36 (30.25%)	53 (35.57%)	
*University graduate*	457 (68.31%)	707 (70.07%)		84 (65.12%)	194 (71.32%)	83 (69.75%)	96 (64.43%)	
**Occupational status, n (%)**			*0.87*					0.48
*Employed*	353 (52.77%)	563 (55.80%)		74 (57.36%)	142 (52.21%)	66 (55.46%)	71 (47.65%)	
*Student*	171 (25.56%)	271 (26.86%)		29 (22.48%)	67 (24.63%)	34 (28.57%)	41 (27.52%)	
*Unemployed*	145 (21.67%)	175 (17.34%)		26 (20.16%)	63 (23.16%)	19 (15.97%)	37 (24.83%)	
**Self-estimated IELT, mintues**	2.40 ± 1.04	4.45 ± 1.72	*<0.001*	1.47 ± 0.72	1.92 ± 0.85	2.9 ± 1.64	3.67 ± 2.28	*<0.001*
**Duration of the sexual relationship, years**	8.82 ± 3.75	8.57 ± 4.02	*0.1*	6.75 ± 3.34	10.83 ± 5.65	7.35 ± 2.28	8.12 ± 4.02	*<0.001*
**Frequency of sexual intercourse in the past four weeks, times**	5.13 ± 3.35	6.54 ± 2.80	*<0.001*	3.85 ± 2.40	5.27 ± 3.02	6.28 ± 3.91	5.06 ± 3.45	*<0.001*

PE = Premature ejaculation; LPE = Lifelong Premature Ejaculation; APE = Acquired Premature Ejaculation; VPE = Variable Premature Ejaculation; SPE = Subjective Premature Ejaculation; BMI = Body Mass Index; IELT = Intra-vaginal Ejaculation Latency Time.

As shown in Table 1, the self-reported PE group was significantly different from the non-PE group in terms of age, BMI, smoking and physical activity ratio, self-reported IELT, and frequency of sexual intercourse over the past 4 weeks (except for *P* = .01 for physical activity, all others were less than 0.001). Specifically, the average age of the PE group was significantly greater than that of the non-PE group, with a higher BMI, and a greater proportion of smokers and those who lacked physical activity. Moreover, the self-reported IELT of the PE group was notably shorter than that of the non-PE group, and the frequency of sexual intercourse over the past 4 weeks was significantly lower.

In the intergroup comparison of the 4 PE subtypes, differences in age, BMI, smoking and exercise ratio, self-reported IELT, duration of sexual relationships, and frequency of sexual intercourse over the past 4 weeks were also statistically significant (all *P* values < .001). The VPE group had the lowest average age, while the APE group had the highest BMI and smoking ratio, and the lowest exercise ratio. The LPE group reported the shortest duration of sexual relationships and the lowest frequency of sexual intercourse over the past 4 weeks, whereas the VPE group reported the longest duration of sexual relationships. These data reveal unique distributions of physiological and behavioral characteristics among different PE subtypes.

### Outcomes of the MBTI and IPE in men with complaints of PE and control group

As shown in Table 2, when comparing the assessment results of self-reported PE and non-PE groups across MBTI dimensions were compared, the trait attributes of dimensions such as Energy, Perceiving, Judging, and Orientation showed statistically significant differences between groups (all *P* values < .001). Patients in the self-reported PE group primarily exhibited the personality traits of Energy-Introversion, Perceiving-Sensing, Judging-Feeling, and Orientation-Perception. These traits may influence men’s perceptions and emotional responses to sexual activity.

**Table 2:** Outcomes of the MBTI and Index of PE in all subjects.

Demographic information	With PE complaints(N = 669)	Without PE complaints(N = 1009)	*P*	LPE (N = 129)	APE (N = 272)	VPE (N = 119)	SPE (N = 149)	*P*
**MBTI**								
***Energy***			*<0.001*					*<0.001*
*Introversion*	413 (61.73%)	426 (42.22%)		95 (73.64%)	142 (52.21%)	72 (60.50%)	104 (69.80%)	
*Extroversion*	256 (38.27%)	583 (57.78%)		34 (26.36%)	130 (47.79%)	47 (39.50%)	45 (30.20%)	
***Perceiving***			*<0.001*					*<0.001*
*Sensing*	403 (60.24%)	493 (48.86%)		103 (79.84%)	135 (49.63%)	74 (62.18%)	91 (61.07%)	
*Intuition*	266 (39.76%)	516 (51.14%)		26 (20.16%)	137 (50.37%)	45 (37.82%)	58 (38.93%)	
***Judging***				*<0.001*				*0.41*
*Feeling*	385 (57.55%)	404 (40.04%)		82 (63.57%)	151 (55.51%)	70 (58.82%)	82 (55.03%)	
*Thinking*	284 (42.45%)	605 (59.96%)		47 (36.43%)	121 (44.49%)	49 (41.18%)	67 (44.97%)	
***Orientation***				*<0.001*				*0.02*
*Perception*	385 (57.55%)	401 (39.74%)		78 (60.47%)	138 (50.74%)	72 (60.50%)	97 (65.10%)	
*Judgment*	284 (42.45%)	608 (60.26%)		51 (39.53%)	134 (49.26%)	47 (39.50%)	52 (34.90%)	
**Index of PE**								
*Total score*	22.01 ± 10.03	-		20.84 ± 9.74	14.99 ± 5.52	27.86 ± 10.65	31.15 ± 14.27	*<0.001*
*Sexual satisfaction*	11.56 ± 4.55	-		9.85 ± 3.82	8.40 ± 3.35	14.74 ± 5.90	16.28 ± 6.21	*<0.001*
*Control over ejaculation*	6.16 ± 3.09	-		6.47 ± 3.01	3.57 ± 1.94	8.15 ± 4.05	9.04 ± 4.36	*<0.001*
*Distress about PE*	4.28 ± 2.07	-		4.52 ± 1.98	3.02 ± 1.47	4.97 ± 2.54	5.83 ± 3.52	*<0.001*

PE = Premature ejaculation; LPE = Lifelong Premature Ejaculation; APE = Acquired Premature Ejaculation; VPE = Variable Premature Ejaculation; SPE = Subjective Premature Ejaculation; MBTI = Myers-Briggs Type Indicator.

### Outcomes of the MBTI and IPE in men with 4 types of PE

A comparison of the evaluation results of the 4 PE subtypes in the MBTI and IPE dimensions revealed that the trait attributes of some dimensions of the MBTI (except Judging) and the total score and scores of each dimension of the IPE were statistically significant among the 4 PE subtypes (Orientation *P* = .02, others *P* < .001).

In terms of energy, the Introversion trait attribute is more prevalent in LPE patients, while Extraversion is more common in APE patients. In terms of survival, the Sensing trait attribute is more likely to be found in LPE patients, whereas Intuition is more common in APE patients. With respect to Orientation, the Perception trait attribute is more common in SPE patients, while Judgment is more common in APE patients. These differences indicate that the psychological traits and physiological behavioral characteristics of PE subtypes are closely related, potentially influencing both sexual function and mental health, providing important evidence for personalized treatment. Additionally, when the IPE assessment results were analyzed, SPE patients scored highest overall and across all dimensions, while LPE patients scored lowest overall and across all dimensions.

### Relationships between the MBTI, self-estimated IELT and index of PE in men with complaints of PE

As shown in Table 3, some personality dimensions of the MBTI are significantly correlated with self-reported IELT and the Index of PE (*P* < .001). Specifically, the Energy-Introversion dimension has the highest correlation coefficient with self-estimated IELT among all dimensions (Adjusted *r* = −0.68, *P* < .001). Moreover, in terms of the relationship between the MBTI and the Index of PE, the Energy-Introversion dimension has the highest correlation coefficient with IPE Distress about PE (Adjusted *r* = 0.74, *P* < .001). In the Perception-Sensation dimension, Perception-Sensation has the highest correlation coefficient with Sexual Satisfaction (Adjusted *r* = 0.70, *P* < .001). In the Orientation-Perception dimension, Orientation-Perception has the highest correlation coefficient with Sexual Satisfaction (Adjusted *r* = 0.64, *P* < .001). We must state that all the *P*-values have been corrected for FDR.

**Table 3:** Relationships between the MBTI, self-estimated IELT and Index of PE in men with complaints of PE.

MBTI			IPE							
	Self-estimated IELT		*Total score*		*Sexual satisfaction*		*Control over ejaculation*		*Distress about PE*	
	*Adjusted r*	*P*	*Adjusted r*	*P*	*Adjusted r*	*P*	*Adjusted r*	*P*	*Adjusted r*	*P*
***Energy-Introversion***	-0.68	*<0.001*	0.60	*<0.001*	0.62	*<0.001*	0.60	*<0.001*	0.74	*<0.001*
***Perceivin-Sensing***	-0.62	*<0.001*	0.64	*<0.001*	0.70	*<0.001*	0.62	*<0.001*	0.65	*<0.001*
***Orientation-Perception***	-0.41	*<0.001*	0.52	*<0.001*	0.64	*<0.001*	0.60	*<0.001*	0.45	*<0.001*

PE = Premature ejaculation; LPE = Lifelong Premature Ejaculation; APE = Acquired Premature Ejaculation; VPE = Variable Premature Ejaculation; SPE = Subjective Premature Ejaculation; IPE = Index of Premature Ejaculation; IELT = Intra-vaginal Ejaculation Latency Time; MBTI = Myers-Briggs Type Indicator.

### The most types of personality assessed by MBTI in PE and its subtypes

As shown in Table 4, among the participants in this study, the ISFP personality type is most common in those with PE. Among the 4 subtypes of PE, the INFP personality type is most common in those with LPE, the ESFJ personality type is most common in those with APE, the ISFP personality type is most common in those with VPE, and the ESFP personality type is most common in those with SPE.

**Table 4:** The most types of personality assessed by MBTI in PE and its subtypes.

Types of PE	Most types of personality
With PE complaints	ISFP
*LPE*	INFP
*APE*	ESFJ
*VPE*	ISFP
*SPE*	ESFP

PE = Premature ejaculation; LPE = Lifelong Premature Ejaculation; APE = Acquired Premature Ejaculation; VPE = Variable Premature Ejaculation; SPE = Subjective Premature Ejaculation; MBTI = Myers-Briggs Type Indicator; I = Introversion; E = Extroversion; S = Sensing; N = Intuition; F = Feeling; P = Perception; J = Judgment.

## Discussion

As the social economy and cultural standards continue to rise, people’s focus on sexual quality has gradually shifted from “physiological satisfaction” to “emotional and experiential optimization,” forming a multi-dimensional demand upgrade. The maturity of personality, the depth of emotional connection, and the harmony of sexual life together form the 3 pillars of intimate relationships. Premature ejaculation is the most prevalent male sexual dysfunction, and significantly impacts the harmony of sexual life. Patients with PE often have mental and psychological problems. Specifically, they may exhibit obvious anxiety or depression.

Personality traits, as inherent attributes of the mental and psychological state, often have complex correlations with the onset of PE. The identikit of the PE-patient comprises the presence of a marked anxiety trait/dysfunction. People with tendencies toward anxiety or neuroticism are more likely to overly focus excessively on sexual performance.^3^ This intensified self-monitoring can disrupt the natural rhythm of sexual responses, leading to decreased ejaculation control.^12^ For example, the requested “sex competence” of the perfectionist personality traits may translate into somatic reactions under high-pressure situations, whereas avoidant attachment patterns, due to emotional suppression or fear of intimacy, may block deep connections through unconscious PE behaviors.^13^ Additionally, internal conflicts in sexual attitudes (such as moral constraints or feelings of shame) if not properly addressed in personality integration, can exacerbate the physical and psychological disconnection in sexual behavior. It is worth noting that PE is not merely a “defect” of personality but a signal of the interaction between body and mind that through cognitive-behavioral interventions or psychodynamic exploration. Individuals can gradually repair their self-acceptance of sex, transforming sensitivity and awareness of the personality trait into empathy for partner needs, thereby reconstructing a more relaxed mode of sexual expression.^14^

Personality traits, as important components of individual psychological characteristics, may play a significant role in the onset of PE. Studying personality traits and their relationships with the onset of PE will help better understand the etiology of PE and provide new intervention strategies for clinical practice, thereby improving patients’ quality of life and sexual relationships. For example, for individuals with lower emotional stability, psychological interventions (such as cognitive-behavioral therapy) can alleviate anxiety, thus reducing the risk of PE or promoting its recovery.^12-15^ Moreover, as an essential part of individual psychology, research findings on personality traits can also provide valuable insights for the development of personalized treatment plans, thereby enhancing the effectiveness of PE treatment.

In the field of psychology, the association between personality traits and sexual health is a complex and multidimensional topic. Personality dimensions may indirectly affect the stability of sexual relationships through psychological mechanisms (such as anxiety, stress coping patterns, behavioral tendencies in intimate relationships).

The results of this study indicate that extroverted men are less likely to suffer from PE. This might be because they are better at socializing to relieve stress, which could lead them to be more confident and relaxed during sexual activities. However, this approach of relying on social interaction to relieve stress may also cause them to neglect their partner’s emotional needs, especially when they are pursuing a sense of “dominance.” This could make the relationship tense. Moreover, extroverted men tend to draw energy from the outside world, and are easily influenced by external environmental stimuli (eg, auditory, visual, etc.) during sexual arousal. This might be the reason why some extroverted men suffer from PE. Furthermore, if a person excessively seeks excitement, they may become overly excited, which can lead to a decline in their ability to control ejaculation.^16^ In stark contrast, introverted men tend to internalize these experiences when facing sexual stimuli, showing deeper and more sensitive emotions. However, this internalization of emotions might be the reason why introverted men are more prone to developing PE. On the one hand, an introverted personality may cause them to pay excessive attention to sexual matters, which may lead to stress when sexual satisfaction is not achieved, making it difficult for them to control ejaculation. On the other hand, the high sensitivity of introverted men may make them more likely to feel pleasure during sexual activities and more easily achieve satisfaction, which may also lead to premature ejaculation.^17^ Additionally, many introverted men, owing to a lack of opportunities to express themselves and be understood, may exhibit excessive self-focus in sexual activities, leading to higher rates of ejaculation and further exacerbating the issue of PE. These personality traits and behavioral patterns may explain why introverted men are more prone to premature ejaculation during sexual activity.

Compared with intuitive men, sensory men tend to focus more on the specific sensory experiences and real-world details of the present moment. This trait may lead to increased sensitivity to physiological stimuli. During sexual activity, they tend to focus on the pleasure derived from physical contact (such as touching, rhythm, etc.), which may lead to excessive excitement of the sympathetic nervous system and potentially result in premature ejaculation.^1^ In contrast, intuitive men tend to rely on abstract associations and future imagination. This cognitive pattern may cause them to place more emphasis on emotional connections or symbolic meanings in sexual interactions,^18^ thereby diverting their attention from physical stimuli and achieving effective buffering. Furthermore, sensory-oriented men tend to prefer standardized procedures.^19^ This may cause them to overly focus on technical details or time control, which can exacerbate psychological stress and create a vicious cycle, while intuitive men’s more flexible thinking style helps them maintain a relaxed state.

Men who focus on both rational analysis and emotional expression may feel anxious due to excessive emphasis on “performance,” but there are fundamental differences in their psychological burdens. People with rich emotions are more likely to overly rely on external emotional feedback (such as the reactions of their partners, and the atmosphere of the relationship),^20^ which may cause them to constantly pay attention to the satisfaction signals from their partners constantly during intimate interactions. This intense need for emotions may lead to the emergence of “appeasement anxiety,” causing one to remain in a state of high alert all the time, and even resulting in unnecessary stress for oneself owing to over interpreting subtle expressions or actions. This might also be a potential cause of the emergence of PE. In contrast, analytical men tend to evaluate sexual performance using objective standards (such as skill application, and physiological control). Their rational attribution model can partially buffer mood swings, allowing them to reconstruct a sense of control through technical strategies (such as rhythm adjustment) even under pressure. Moreover, the natural tendency of emotional men to avoid conflict may suppress communication needs,^21^ preventing potential psychological tension from being alleviated through dialog, whereas analytical men are more likely to objectify problems for analysis, which forms psychological protection. This different approach may affect the physiological regulation of their ejaculation.

Men with perceptual traits are more likely to suffer from PE than those with critical traits. This might be due to differences in self-regulation.. Both can experience PE due to imbalanced stress regulation, but perceptual men are more susceptible to dynamic environmental disturbances. They prefer flexible and open behavioral modes, which may lead them to overfocus on situational changes (such as atmosphere fluctuations, sudden stimuli) during intimate interactions, thus diverting their attention from their own physiological responses. This distraction of attention may weaken their ability to control ejaculation.^22^ Moreover, the “instant feedback” characteristic of emotional men may cause them to pursue pleasure too early without considering the rhythm, which might be the reason why they are more prone to PE, whereas rational men are better at skills such as regular breathing or muscle control to maintain stability.^23^ Furthermore, compared with analytical men, intuitive men are more prone to experiencing emotional fluctuations when facing unexpected situations, which might affect their ability to control ejaculation.

Introverted males may face greater stress in their sexual life, and this phenomenon is likely primarily associated with the interaction between their internal psychological mechanisms and external situational factors. From the perspective of psychological traits, introverts typically tend to engage in deep introspection and emotional internalization, they may be more sensitive to self-expression and partner feedback, and could potentially get bogged down in excessive rumination about details during sexual interactions (eg, concerns about physical attractiveness, sexual performance, or the sufficiency of emotional connection with their partner).^24^ This additional cognitive load may, to some extent, weaken their ability to engage naturally in sexual experiences. Meanwhile, introverts may often require more time to establish trust and emotional security. If there are communication barriers in sexual interactions, or if clear emotional resonance fails to form between partners, they may develop anxiety due to their inability to adapt quickly to such interactive dynamics. Furthermore, the trait of social energy depletion in introverts means they are usually more likely to need solitude to recover their energy after intimate contact. If partners fail to understand and respect this need, it may further exacerbate the stress caused by mismatched expectations. The combination of these factors may collectively pose unique challenges for individuals with introverted personalities in sexual interactions. From a neurophysiology perspective, existing research suggests that introverts may have relatively lower sensitivity to dopamine (associated with external rewards and stimulation-seeking) and may exhibit higher dependence on cholinergic neural pathways (associated with internal cognitive processing and calm states).^25^ The high arousal state during sexual interactions may potentially exceed their “optimal stimulation threshold,” thereby triggering avoidance tendencies. Prolonged exposure to overly stimulating contexts may lead to elevated cortisol levels, which in turn intensifies stress perception.

In the assessment of sexual satisfaction, sensory men tend to have lower scores.. This might be due to their reliance on specific sensory inputs and the immediate feedback from real-life details.^26^ Therefore, it may result in the situation where when the interaction between partners lacks specific physical stimulation (such as rich tactile or visual experiences) or comfortable environmental conditions (such as temperature or noise disturbances), their attention is easily distracted, thereby causing the experience to become fragmented. Additionally, their dependence on clear procedures and predictability may cause cognitive dissonance when spontaneous changes occur during sexual activity. Furthermore, if the 2 parties fail to reach an agreement on the pace and intensity, the gap between the actual experience and the ideal state will further reduce the satisfaction of both parties, and this may also lower their sexual capabilities.

Men with highly sensitive senses tend to have a lower threshold for sexual arousal,^27^ and strong immediate feedback can accelerate the process of physiological arousal. At the same time, excessive focus on specific movements or environmental details (such as physical sensations, precise rhythms) may consume cognitive resources, weakening their awareness and regulation of their own body responses (such as breathing, muscle tension). Furthermore, the demand for “process controllability” may trigger anxiety. When sexual activity deviates from the predetermined rhythm, it may easily cause tension, which may further intensify the physiological response and lead to premature ejaculation.^12^ These reasons might be the reasons why sensory men are more prone to PE. Therefore, improving ejaculation control requires starting with cognitive restructuring, such as through sensory and emotional balance training, enhancing immersion in the overall experience rather than fixating on local details, and combining relaxation techniques to reduce excessive vigilance towards uncontrollable factors.

In subsequent research, we explored the association between specific personality types and PE subtypes, which will provide theoretical clues for precise intervention. In our study, it was observed that individuals with INFP personality traits were relatively more common among lifelong PE patients. This phenomenon may be closely related to the interaction between their unique psychological traits and physiological mechanisms. INFP individuals typically exhibit high sensitivity, introspective tendencies, and a deep pursuit of ideal relationships, which can translate into excessive self-examination and anxiety in sexual behavior.^28^ Specifically, their expectations regarding sexual experiences are usually quite high. However, this can lead to a vicious cycle: “Expectations unmet → Anxiety intensifies → Loss of control,” which may subsequently lower the threshold for ejaculation. Meanwhile, people of the INFP type tend to internalize their emotions rather than express them actively.^29^ This might affect the balance of neurotransmitters (such as serotonin), which is crucial for controlling ejaculation, thereby potentially impacting the regulation ability of ejaculation. Furthermore, they usually have a strong need for emotional compatibility. However, if there are differences in the real relationship, it may lead to avoidance or resistance towards sexual behavior, which might be a potential cause for their having PE.

The high proportion of ESFJ (Extraverted, Sensing, Feeling, Judging) males in the acquired PE group may be closely related to their unique psychological-neurological interaction patterns driven by personality traits. An extroverted emotional (E) personality may cause them to overly focus on the immediate feedback from their partner (such as the frequency of sexual climax, body language or emotional fluctuations), equating sexual performance to the degree of “relationship responsibility” fulfilled. This may lead to anxiety and thereby affect the regulation of ejaculation.^30^ Meanwhile, the introverted perception (S) often makes them recall failed experiences. When facing similar scenarios, anxiety will quickly arise, resulting in a decline in the regulation ability to regulate ejaculation.^31^

Research findings indicate that the ISFP personality type may be associated with variant PE. This type of person has 2 conflicting traits: on the one hand, they intensely pursue a sense of “authenticity” in sexual encounters (such as demanding emotional purity), while on the other hand, they are exceptionally sensitive to sudden changes. They capture bodily details like a real-time scanner (such as touch intensity, breathing rate). However, due to their lack of rational analysis (like telling themselves “these reactions are normal”), anxiety accumulates, leading to prolonged tension in the body. More uniquely, although they have strong adaptability, sudden changes can disrupt the rhythm. This disruption of rhythm and the emergence of anxiety may interfere with the body’s ability to control ejaculation, ultimately resulting in significant fluctuations in the timing of ejaculation—this abnormal pattern caused by psychological fluctuations is significantly different from the traditional physiological factors that lead to premature ejaculation.

The potential association between the ESFP type (Extraversion, Sensing, Emotional, Perceiving) and subjective PE (where the patient’s subjective anxiety is significant while the objective latency for ejaculation is normal) may stem from the interaction between the immediate feedback dependency driven by the core trait “extraversion-sensing” and the evaluative sensitivity along the “emotional-perceiving” axis. These people are similar to those who are extremely focused on sensory stimulation. Their relentless pursuit of “perfect performance” can magnify poor performance.^11^ In sexual behavior, these individuals pay close attention to sensory details (such as visual stimulation and tactile intensity), and their insistence on “perfect performance” can exaggerate normal variations (such as occasional shorter durations) into evidence of “sexual dysfunction.” This excessive sensitivity to details and overemphasis on them may cause them to become overly anxious, thereby affecting their sexual function.

We discussed the association between all types of personality traits and PE based on the MBTI. However, we did not explore the association of alexithymia with PE in this study. Alexithymia refers to affective deficits in differentiating, identifying, and communicating one’s feelings. We should first consider the situation rather than the personality traits. A relevant study demonstrated that alexithymic features, particularly an externally oriented cognitive style, can be seen as possible risk and/or maintenance factors for PE, and may contribute to a more serious manifestation of this condition.^32^

Although some studies have explored the relationship between personality traits and PE, existing research still has certain limitations. First, the primary causes of PE often involve physiological factors (such as genetics, hormones, and nerve sensitivity) or specific psychological traumas (such as sexual shame and childhood experiences), with personality traits only indirectly influencing PE through mental states. A systematic framework for personality assessment can help individuals and organizations enhance self-awareness and team collaboration, but personality types are not fixed. They can change due to the influence of self-awareness changes over time and questionnaire completion attitudes. Therefore, sexual health issues need to be analyzed comprehensively in the context of specific situations (such as partner relationships and cultural backgrounds). Second, the sample size was insufficient and concentrated in Anhui Province. Third, the research methods are primarily cross-sectional surveys, making it difficult to establish a causal relationship between personality traits and PE. Fourth, given that our research was conducted in a single province of China, and that culture may influence a person’s personality and attitude towards PE, the results may not be universally applicable. We need to conduct more research in more countries and regions. Fifth, our assessment of PE is mainly based on self-reports and IPE, but this method has the potential for recall errors, which can lead to unreliable results. Finally, we explored the relationship between personality and PE on the basis of only a single personality trait scale. The assessment methods that we should use with other personality theories should be used to conduct the same study to understand the relationship between personality and PE. Moreover, we must acknowledge the most significant drawback, which is that the MBTI has undergone numerous changes and has relatively poor repeatability, thereby weakening the robustness of the results. Meanwhile, The MBTI is currently widely used in career assessments. It is less frequently utilized in some scientific studies. However, these measuring tools hold significant importance in the exploration of personality traits. Our study aims to help patients with different personality traits better address the PE and have a better attitude toward it. Considering the variability of personality traits, more research is needed to confirm our findings in the future. Furthermore, the underlying mechanisms linking personality traits to PE have not been fully explored. Therefore, it is necessary to conduct larger-scale, more rigorous community-based studies to further elucidate the role of personality traits in the development of PE.

## Conclusion

Men with traits of Introversion, Sensing, Feeling, and Perception are more common in the population with PE, therefore, people with these personality traits, we need more attention. The INFP personality type is more common in those with LPE, the ESFJ personality type is more common in those with APE, the ISFP personality type is more common in those with VPE, and the ESFP personality type is more common in those with SPE. Therefore, the findings of this study suggest that personality trait factors may influence the pathogenesis of PE, and the use of the MBTI personality assessment provides a new perspective on how personality traits play a role in the onset of PE. In addition, considering that male PE patients with different personality traits have different attitudes toward the treatment of PE, individualized programs for PE patients should be reconsidered. Further studies are needed to confirm and extend these results.

## Supplementary Material

9-15_Figure_1_qfaf094_qfaf094

Table_1_qfaf094

Table_2_qfaf094

Table_3_qfaf094

Table_4_qfaf094
