# Supplementary figures and images for: Study of psychosocial factors affecting premature ejaculation from the perspective of personality traits: a large sample cross-sectional study from Anhui, China
Source: Sex Med. 2025 Nov 15;13(5):qfaf094. doi: 10.1093/sexmed/qfaf094 (PMC12619530; doi:10.1093/sexmed/qfaf094)

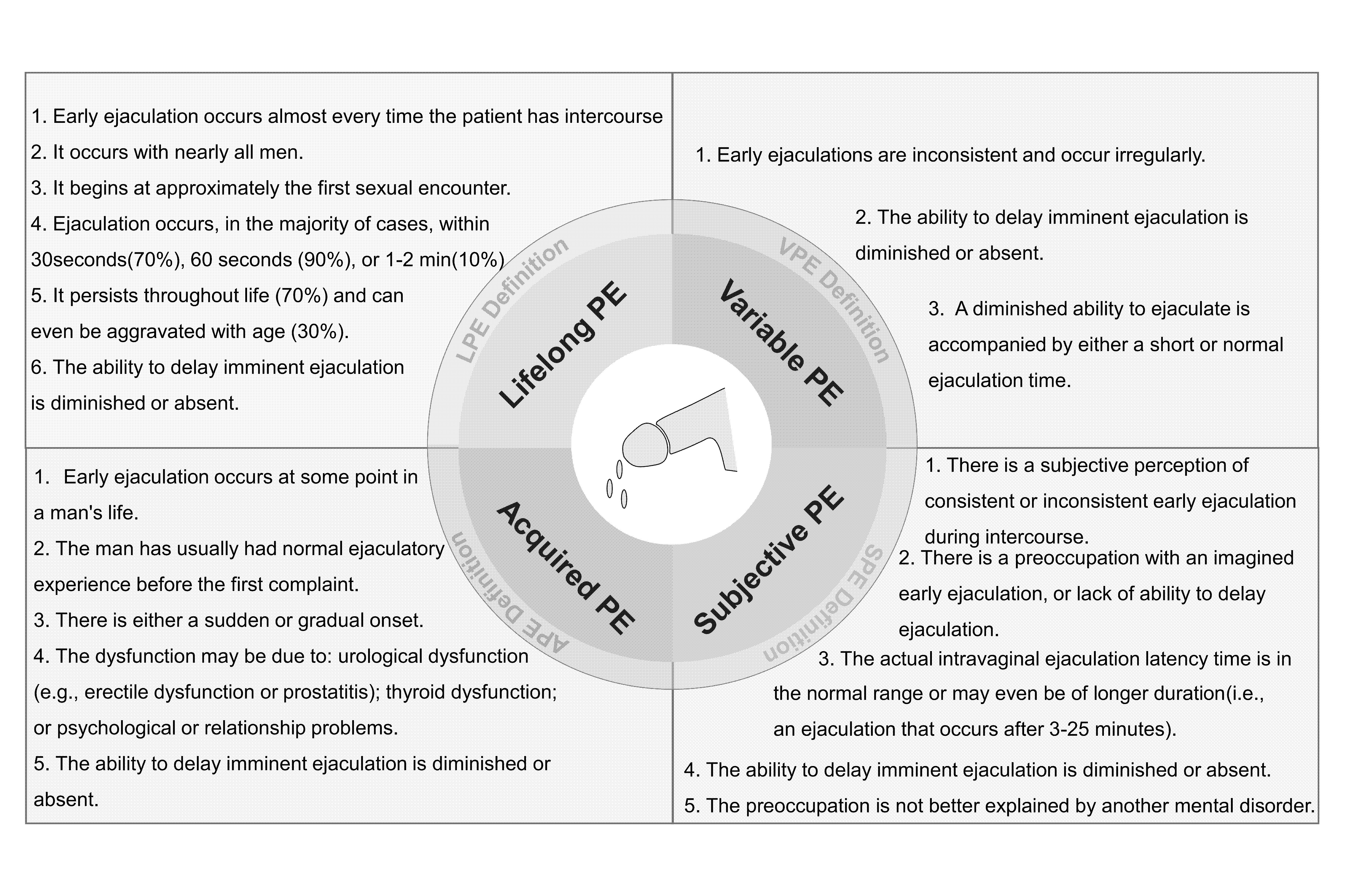

Supplement: 9-15_Figure_1_qfaf094_qfaf094 [file 9-15_figure_1_qfaf094_qfaf094.jpeg]
