# Supplementary material for: Study of psychosocial factors affecting premature ejaculation from the perspective of personality traits: a large sample cross-sectional study from Anhui, China
Source: Sex Med. 2025 Nov 15;13(5):qfaf094. doi: 10.1093/sexmed/qfaf094 (PMC12619530; doi:10.1093/sexmed/qfaf094)
Supplement: Table_1_qfaf094 [file table_1_qfaf094.doc]

| **Table 1. Demographic information of males with and without PE complaints** | | | | | | | | | | | | | | | | | | | | | | | | | | |
| --- | --- | --- | --- | --- | --- | --- | --- | --- | --- | --- | --- | --- | --- | --- | --- | --- | --- | --- | --- | --- | --- | --- | --- | --- | --- | --- |
| **Demographic information** | **With PE complaints（N=669）** | | | | **Without PE complaints（N=1009）** | | | | ***P^1^*** | **LPE**  **（N=129）** | | | | **APE**  **（N=272）** | | |  | **VPE**  **（N=119）** | | |  | **SPE**  **（N=149）** | | |  | ***P^2^*** |
| **Age, years** | 40.72 | ± | 13.36 |  | 33.37 | ± | 15.75 |  | *<0.001* | 35.82 | ± | 12.87 |  | 47.75 | ± | 15.33 |  | 32.29 | ± | 11.02 |  | 38.84 | ± | 10.42 |  | *<0.001* |
| **BMI score, kg/m2** | 24.38 | ± | 4.35 |  | 22.56 | ± | 3.85 |  | *<0.001* | 23.35 | ± | 4.02 |  | 25.82 | ± | 3.91 |  | 23.47 | ± | 4.15 |  | 23.37 | ± | 3.82 |  | *<0.001* |
| **Smoking, n(%)** | 402 | （ | 60.09% | ） | 471 | （ | 46.68% | ） | *<0.001* | 77 | （ | 59.69% | ） | 197 | （ | 72.43% | ） | 63 | （ | 52.94% | ） | 65 | （ | 43.62% | ） | *<0.001* |
| **Exercise, n(%)** | 258 | （ | 38.57% | ） | 437 | （ | 43.31% | ） | *0.01* | 50 | （ | 38.76% | ） | 78 | （ | 28.68% | ） | 56 | （ | 47.06% | ） | 74 | （ | 49.66% | ） | *<0.001* |
| **Educational status, n(%)** |  |  |  |  |  |  |  |  | *0.44* |  |  |  |  |  |  |  |  |  |  |  |  |  |  |  |  | *0.41* |
| *High school or less* | 212 | （ | 31.69% | ） | 302 | （ | 29.93% | ） |  | 45 | （ | 34.88% | ） | 78 | （ | 28.68% | ） | 36 | （ | 30.25% | ） | 53 | （ | 35.57% | ） |  |
| *University graduate* | 457 | （ | 68.31% | ） | 707 | （ | 70.07% | ） |  | 84 | （ | 65.12% | ） | 194 | （ | 71.32% | ） | 83 | （ | 69.75% | ） | 96 | （ | 64.43% | ） |  |
| **Occupational status, n(%)** |  |  |  |  |  |  |  |  | *0.87* |  |  |  |  |  |  |  |  |  |  |  |  |  |  |  |  | 0.48 |
| *Employed* | 353 | （ | 52.77% | ） | 563 | （ | 55.80% | ） |  | 74 | （ | 57.36% | ） | 142 | （ | 52.21% | ） | 66 | （ | 55.46% | ） | 71 | （ | 47.65% | ） |  |
| *Student* | 171 | （ | 25.56% | ） | 271 | （ | 26.86% | ） |  | 29 | （ | 22.48% | ） | 67 | （ | 24.63% | ） | 34 | （ | 28.57% | ） | 41 | （ | 27.52% | ） |  |
| *Unemployed* | 145 | （ | 21.67% | ） | 175 | （ | 17.34% | ） |  | 26 | （ | 20.16% | ） | 63 | （ | 23.16% | ） | 19 | （ | 15.97% | ） | 37 | （ | 24.83% | ） |  |
| **Self-estimated IELT, mintues** | 2.40 | ± | 1.04 |  | 4.45 | ± | 1.72 |  | *<0.001* | 1.47 | ± | 0.72 |  | 1.92 | ± | 0.85 |  | 2.9 | ± | 1.64 |  | 3.67 | ± | 2.28 |  | *<0.001* |
| **Duration of the sexual relationship, years** | 8.82 | ± | 3.75 |  | 8.57 | ± | 4.02 |  | *0.1* | 6.75 | ± | 3.34 |  | 10.83 | ± | 5.65 |  | 7.35 | ± | 2.28 |  | 8.12 | ± | 4.02 |  | *<0.001* |
| **Frequency of sexual intercourse in the past four weeks, times** | 5.13 | ± | 3.35 |  | 6.54 | ± | 2.80 |  | *<0.001* | 3.85 | ± | 2.40 |  | 5.27 | ± | 3.02 |  | 6.28 | ± | 3.91 |  | 5.06 | ± | 3.45 |  | *<0.001* |

PE=Premature ejaculation; LPE=Lifelong Premature Ejaculation; APE=Acquired Premature Ejaculation; VPE=Variable Premature Ejaculation; SPE=Subjective Premature Ejaculation

BMI=Body Mass Index; IELT=Intra-vaginal Ejaculation Latency Time;
