# Supplementary material for: Study of psychosocial factors affecting premature ejaculation from the perspective of personality traits: a large sample cross-sectional study from Anhui, China
Source: Sex Med. 2025 Nov 15;13(5):qfaf094. doi: 10.1093/sexmed/qfaf094 (PMC12619530; doi:10.1093/sexmed/qfaf094)
Supplement: Table_2_qfaf094 [file table_2_qfaf094.doc]

| **Table 2. Outcomes of the MBTI and Index of PE in all subjects** | | | | | | | | | | | | | | | | | | | | | | | | | | |
| --- | --- | --- | --- | --- | --- | --- | --- | --- | --- | --- | --- | --- | --- | --- | --- | --- | --- | --- | --- | --- | --- | --- | --- | --- | --- | --- |
| **Demographic information** | **With PE complaints（N=669）** | | | | **Without PE complaints（N=1009）** | | | | ***P*** | **LPE**  **（N=129）** | | | | **APE**  **（N=272）** | | |  | **VPE**  **（N=119）** | | |  | **SPE**  **（N=149）** | | |  | ***P*** |
| **MBTI** |  |  |  |  |  |  |  |  |  |  |  |  |  |  |  |  |  |  |  |  |  |  |  |  |  |  |
| ***Energy*** |  |  |  |  |  |  |  |  | *<0.001* |  |  |  |  |  |  |  |  |  |  |  |  |  |  |  |  | *<0.001* |
| *Introversion* | 413 | （ | 61.73% | ） | 426 | （ | 42.22% | ） |  | 95 | （ | 73.64% | ） | 142 | （ | 52.21% | ） | 72 | （ | 60.50% | ） | 104 | （ | 69.80% | ） |  |
| *Extroversion* | 256 | （ | 38.27% | ） | 583 | （ | 57.78% | ） |  | 34 | （ | 26.36% | ） | 130 | （ | 47.79% | ） | 47 | （ | 39.50% | ） | 45 | （ | 30.20% | ） |  |
| ***Perceiving*** |  |  |  |  |  |  |  |  | *<0.001* |  |  |  |  |  |  |  |  |  |  |  |  |  |  |  |  | *<0.001* |
| *Sensing* | 403 | （ | 60.24% | ） | 493 | （ | 48.86% | ） |  | 103 | （ | 79.84% | ） | 135 | （ | 49.63% | ） | 74 | （ | 62.18% | ） | 91 | （ | 61.07% | ） |  |
| *Intuition* | 266 | （ | 39.76% | ） | 516 | （ | 51.14% | ） |  | 26 | （ | 20.16% | ） | 137 | （ | 50.37% | ） | 45 | （ | 37.82% | ） | 58 | （ | 38.93% | ） |  |
| ***Judging*** |  |  |  |  |  |  |  |  | *<0.001* |  |  |  |  |  |  |  |  |  |  |  |  |  |  |  |  | *0.41* |
| *Feeling* | 385 | （ | 57.55% | ） | 404 | （ | 40.04% | ） |  | 82 | （ | 63.57% | ） | 151 | （ | 55.51% | ） | 70 | （ | 58.82% | ） | 82 | （ | 55.03% | ） |  |
| *Thinking* | 284 | （ | 42.45% | ） | 605 | （ | 59.96% | ） |  | 47 | （ | 36.43% | ） | 121 | （ | 44.49% | ） | 49 | （ | 41.18% | ） | 67 | （ | 44.97% | ） |  |
| ***Orientation*** |  |  |  |  |  |  |  |  | *<0.001* |  |  |  |  |  |  |  |  |  |  |  |  |  |  |  |  | *0.02* |
| *Perception* | 385 | （ | 57.55% | ） | 401 | （ | 39.74% | ） |  | 78 | （ | 60.47% | ） | 138 | （ | 50.74% | ） | 72 | （ | 60.50% | ） | 97 | （ | 65.10% | ） |  |
| *Judgment* | 284 | （ | 42.45% | ） | 608 | （ | 60.26% | ） |  | 51 | （ | 39.53% | ） | 134 | （ | 49.26% | ） | 47 | （ | 39.50% | ） | 52 | （ | 34.90% | ） |  |
| **Index of PE** |  |  |  |  |  |  |  |  |  |  |  |  |  |  |  |  |  |  |  |  |  |  |  |  |  |  |
| *Total score* | 22.01 | ± | 10.03 |  | - |  | - |  |  | 20.84 | ± | 9.74 |  | 14.99 | ± | 5.52 |  | 27.86 | ± | 10.65 |  | 31.15 | ± | 14.27 |  | *<0.001* |
| *Sexual satisfaction* | 11.56 | ± | 4.55 |  | - |  | - |  |  | 9.85 | ± | 3.82 |  | 8.40 | ± | 3.35 |  | 14.74 | ± | 5.90 |  | 16.28 | ± | 6.21 |  | *<0.001* |
| *Control over ejaculation* | 6.16 | ± | 3.09 |  | - |  | - |  |  | 6.47 | ± | 3.01 |  | 3.57 | ± | 1.94 |  | 8.15 | ± | 4.05 |  | 9.04 | ± | 4.36 |  | *<0.001* |
| *Distress about PE* | 4.28 | ± | 2.07 |  | - |  | - |  |  | 4.52 | ± | 1.98 |  | 3.02 | ± | 1.47 |  | 4.97 | ± | 2.54 |  | 5.83 | ± | 3.52 |  | *<0.001* |

PE=Premature ejaculation; LPE=Lifelong Premature Ejaculation; APE=Acquired Premature Ejaculation; VPE=Variable Premature Ejaculation; SPE=Subjective Premature Ejaculation

MBTI=Myers-Briggs Type Indicator;
