# Supplementary material for: Study of psychosocial factors affecting premature ejaculation from the perspective of personality traits: a large sample cross-sectional study from Anhui, China
Source: Sex Med. 2025 Nov 15;13(5):qfaf094. doi: 10.1093/sexmed/qfaf094 (PMC12619530; doi:10.1093/sexmed/qfaf094)
Supplement: Table_3_qfaf094 [file table_3_qfaf094.doc]

| **Table 3. Relationships between the MBTI, self-estimated IELT and Index of PE in men with complaints of PE** | | | | | | | | | | |
| --- | --- | --- | --- | --- | --- | --- | --- | --- | --- | --- |
| **MBTI** | **Self-estimated IELT** | | **IPE** | | | | | | | |
|  |  |  | ***Total score*** | | ***Sexual satisfaction*** | | ***Control over ejaculation*** | | ***Distress about PE*** | |
|  | ***Adjusted r*** | ***P*** | ***Adjusted r*** | ***P*** | ***Adjusted r*** | ***P*** | ***Adjusted r*** | ***P*** | ***Adjusted r*** | ***P*** |
| ***Energy-Introversion*** | -0.68 | *<0.001* | 0.60 | *<0.001* | 0.62 | *<0.001* | 0.60 | *<0.001* | 0.74 | *<0.001* |
| ***Perceivin-Sensing*** | -0.62 | *<0.001* | 0.64 | *<0.001* | 0.70 | *<0.001* | 0.62 | *<0.001* | 0.65 | *<0.001* |
| ***Orientation-Perception*** | -0.41 | *<0.001* | 0.52 | *<0.001* | 0.64 | *<0.001* | 0.60 | *<0.001* | 0.45 | *<0.001* |
| PE=Premature ejaculation; LPE=Lifelong Premature Ejaculation; APE=Acquired Premature Ejaculation; VPE=Variable Premature Ejaculation; SPE=Subjective Premature Ejaculation; IPE=Index of Premature Ejaculation;  IELT=Intra-vaginal Ejaculation Latency Time; MBTI=Myers-Briggs Type Indicator; | | | | | | | | | | |
