# Supplementary material for: Study of psychosocial factors affecting premature ejaculation from the perspective of personality traits: a large sample cross-sectional study from Anhui, China
Source: Sex Med. 2025 Nov 15;13(5):qfaf094. doi: 10.1093/sexmed/qfaf094 (PMC12619530; doi:10.1093/sexmed/qfaf094)
Supplement: Table_4_qfaf094 [file table_4_qfaf094.doc]

| **Table 4. The most types of personality assessed by MBTI in PE and its subtypes** | |
| --- | --- |
| **Types of PE** | **Most types of personality** |
| With PE complaints | ISFP |
| *LPE* | INFP |
| *APE* | ESFJ |
| *VPE* | ISFP |
| *SPE* | ESFP |
| PE=Premature ejaculation; LPE=Lifelong Premature Ejaculation; APE=Acquired Premature Ejaculation; VPE=Variable Premature Ejaculation; SPE=Subjective Premature Ejaculation  MBTI=Myers-Briggs Type Indicator;  I=Introversion; E=Extroversion; S=Sensing; N=Intuition; F=Feeling; P=Perception; J=Judgment; | |
